# Collective Statement Regarding Patient Access to Approved Therapies from the Center Directors of Parent Project Muscular Dystrophy’s Certified Duchenne Care Centers

**DOI:** 10.1371/currents.md.4a12c57a46a24603cb3d36d7fe0668b6

**Published:** 2018-03-15

**Authors:** Cristian Ionita, Kathi Kinnett, Katherine Mathews

**Affiliations:** Department of Pediatrics (Neurology), Yale University School of Medicine, New Haven, CT, USA; Parent Project Muscular Dystrophy, MIddletown, OH, USA; Departments of Pediatrics and Neurology, University of Iowa Carver College of Medicine, Iowa City, IA, USA

## Abstract

The dystrophinopathies (Duchenne [DMD] and Becker muscular dystrophy) are progressive diseases that until recently had no specific treatments. New FDA pathways to drug approval in rare diseases have resulted in a dramatic increase in the number of treatment trials for DMD and recently, two approved drugs. Health insurance policies for DMD products have been constructed with limited input from neuromuscular specialists directly involved in patient care and without patient input. These policies often reflect a lack of understanding of the disease, clinical population or the treatment. To ensure that policy determinations reflect best clinical practice, we recommend insurers work with neuromuscular specialists with expertise in care for patients with dystrophinopathy, as well as patients and families, and prominent advocacy organizations, such as Parent Project Muscular Dystrophy, in developing policies.

## Introduction

The FDA recently approved two drugs for treatment of Duchenne muscular dystrophy (DMD). There are many ongoing treatment trials for DMD and it is likely that the treatment options will continue to grow. Recent medical and pharmacy benefit policies from health insurers reviewing DMD products have been written with limited input from neuromuscular specialists directly involved in patient care, and without patient input. This has resulted in excessive variability between payors and a high rate of denials, even for patients who received initial approval and have begun treatment. This has been frustrating for patients, parents, and neuromuscular specialists, and these policies may delay treatment resulting in irreversible disease progression. We therefore urge healthcare insurers to work collaboratively with DMD clinical experts when developing policy determinations.

In 2014 Parent Project Muscular Dystrophy (PPMD) began an effort to identify and certify neuromuscular centers around the United States capable of providing comprehensive care to patients and families living with Duchenne muscular dystrophy (DMD). These centers provide care consistent with the published DMD Care Considerations developed with the support of the Centers for Disease Control (CDC). There are currently 17 of these Certified Duchenne Care Centers (CDCCs) throughout the USA. Each CDCC is directed by a neuromuscular specialist with extensive experience and expertise in the management of DMD. The following statement is intended to reflect the CDCC center directors’ collective position regarding the importance of ensuring that patients with DMD have access to FDA approved therapies when prescribed by their clinicians.

## About Duchenne Muscular Dystrophy

DMD is the most common, lethal neuromuscular disease of childhood with an incidence of 1:5364 males in the US^1^. DMD leads to progressive muscle weakness, and eventual respiratory and cardiac failure. After early years of normal development, there is a noticeable lack of gross motor development, then deterioration of the ability to run and climb stairs. The mean age of diagnosis in the U.S. is 4 years old^2^^,^^3^ . Between the ages of nine and 14, the ability to walk is typically lost. In the late teenage years, respiratory and cardiac function deteriorates. Based upon improvements in care including corticosteroid use, the natural history of the disease has been improved, and many people with Duchenne are now expected to live into their mid to late twenties^4^ .

DMD is caused by mutations in the dystrophin gene on the X-chromosome. This is among the largest genes in the human genome, encompassing 79 exons^5^ . Different types of mutations are encountered including large deletions (60-65%), duplications (5-10%) and point mutations, small deletions, or point mutations/splice site mutations/intronic mutations (25-35%)^6^. These mutations result in a loss of the dystrophin protein. Dystrophin is a key component of the dystrophin-glycoprotein complex (DGC) that creates an essential link between the cytoskeleton and extracellular matrix, critical to maintaining muscle membrane stability and prevent muscle fiber breakdown. The loss of dystrophin results in breakdown of the muscle membrane, reduced resistance to contraction and ultimately muscle fiber death. In addition, non-mechanical roles of dystrophin and other components of the DGC are becoming apparent^7^. Disturbed signaling, as well as regenerative and fibrotic processes likely play a role in downstream pathophysiology and are partly responsible for phenotypic variability^8^. Until recently, no treatment was available to restore dystrophin production.

DMD is one of two diagnoses classified as “dystrophinopathies” (i.e., resulting in a deficiency or abnormality of dystrophin). There is a spectrum of clinical severity with DMD at the more severe end and Becker muscular dystrophy (BMD) demonstrating a milder phenotype. Different mutations in the dystrophin gene can result in complete absence of dystrophin protein, reduction in the amount of protein, or change in function of the protein. In general, patients with complete absence of dystrophin have DMD while those with residual dystrophin have milder phenotypes.

Since the gene was cloned and the dystrophin protein identified three decades ago^9^, a wealth of knowledge has accumulated about disease pathophysiology. Multiple drugs are at different stages of development addressing dystrophin restoration or different pathophysiologic processes involved.

## The Path to Duchenne Therapies

Drug discovery for rare diseases is complicated by the high cost of drug development and small target patient populations. Since the 1980’s, several targeted legislative measures have been implemented to address these challenges in orphan drug development. As a result, the development of orphan drugs became a more sustainable business model for investors and pharmaceutical companies. In 2016 alone, 9 of the 22 novel drugs approved by FDA were to treat orphan diseases. The orphan product space was further seeded through the creation of innovation incentives such as ‘Fast Track’, ‘Break Through’, and Priority Review designations, and the expansion of the Accelerated Approval pathway. The Accelerated Approval pathway was initially available for serious and life-threatening diseases (i.e. HIV) and was expanded in the 2012 Food and Drug Administration Safety and Innovation Act (FDASIA) reauthorization to also include rare diseases. This has led to the Accelerated Approval of a number of new drugs based on surrogate endpoints. The FDA approval of a drug under the Accelerated Approval process is a full approval based on the fact that the surrogate endpoint is “reasonably likely” to predict clinical benefit. The FDA approval of a drug under the Accelerated Approval process mandates that the sponsor pursue clinical trials to further demonstrate the clinical efficacy and safety of their product. The FDA can withdraw approval for several reasons, including failure to demonstrate clinical benefit in follow on studies.

In September 2016, the DMD community celebrated the first FDA approved drug for DMD, Exondys51 (eteplirsen). This was followed by the approval of Emflaza (deflazacort), a corticosteroid that alters the course of DMD, in February 2017.

## Access to Duchenne muscular dystrophy approved therapies

With the approval of these medications and anticipated approval of new therapies in the future, access to treatment has become more complex for the Duchenne community. We acknowledge the high price of these medications, while the US continues to struggle with rising healthcare costs. We also recognize that with the current global system of drug development, bringing an orphan drug to market is expensive. Perhaps as a result of these high costs, payors have developed policies for therapy initiation and continuation often based on incomplete understanding of the natural history and disease progression, potential benefits of these drugs and the risks of withholding therapies. In addition, several insurers have declined to cover these approved therapies, considering them investigational, not medically necessary, or instituting nearly insurmountable barriers to access or prior authorizations that may contradict provider guidance. We, as representatives of the PPMD Certified Duchenne Care Centers and the Duchenne community are extremely troubled by these policies.

It is our view that health insurance should not restrict access to care deemed appropriate by clinicians caring for those lives covered by those health insurers. The role of payors is not to diagnose, determine appropriateness of prescribed therapies, or to contradict the medical opinion of qualified expert medical providers, particularly when use of a drug is “on-label.” To that end, we assert the following positions:

- Coverage of a drug must reflect the FDA approval status of a drug.

- When the FDA determines approval of an investigational agent based on the Accelerated Approval pathway, it becomes an FDA-approved drug. With Accelerated Approval (or any other FDA approval), a therapy is approved and no longer considered an investigational product. The drug should, therefore, no longer be denied as an investigational drug.
- Some insurers, as criteria for renewed approval, have proposed measurement of dystrophin in serial muscle biopsies as criteria for renewed approvals. A muscle biopsy is an invasive surgical procedure requiring sedation and differing degrees of intraoperative ventilatory support. Quantitative measurement of dystrophin protein in a muscle biopsy requires specialized expertise not available in a clinical setting. The risks of a muscle biopsy are carefully considered when constructing clinical trial protocols and may be acceptable for clinical trials, but are not appropriate in broad clinical practice. People living with Duchenne are at increased risk for developing rhabdomyolysis (massive muscle breakdown) and experiencing pulmonary and cardiac failure in the setting of surgical procedures involving general anesthesia. Therefore, this requirement is unreasonable, unethical and impractical.
- Many insurers are utilizing clinical trial outcome measures in order to establish therapeutic efficacy, demonstrating a lack of understanding of this disease. As an example, the 6MWT (6 minute timed walk test) is a research tool often used in clinical trials but not suitable for assessment in clinical practice. Appropriate monitoring parameters for any drug are found in that drug’s United States product insert, which was constructed based on the parameters of the FDA approval.
- While patients may receive a genetic report (genotype) that is consistent with Becker muscular dystrophy, they may exhibit the symptoms (phenotype) of a patient with Duchenne muscular dystrophy. Duchenne and Becker phenotypes are determined clinically as the genetic results are not 100% predictive and the genotype-phenotype correlation is often unpredictable.
- We would therefore endorse that all patients with a genetically confirmed dystrophinopathy, whose neuromuscular specialist feels a therapy is appropriate, have access to that prescribed therapy, regardless of age, or sex.

## Conclusion

Recent health insurance policies for DMD products have been constructed with limited input from neuromuscular specialists directly involved in patient care and without patient input. In order to ensure that policy determinations reflect best clinical practice, we implore insurers to work with neuromuscular specialists leading the PPMD Certified Duchenne Care Center teams, as well as patients and families, in developing policies. We represent a collective body of clinicians and clinical investigators, leading the world’s DMD care, registries, clinical trials, research, and natural history studies. We are committed to data-driven, evidence-based determinations and implementation of longitudinal tracking of outcomes of patients exposed to therapy. We also recommend that prominent advocacy organizations, such as PPMD, be involved in the development of policy determinations. Together, we are eager to engage with payors to develop policies that will improve health outcomes for patients with DMD.

## Corresponding Author

Kathi Kinnett, Parent Project Muscular Dystrophy (kathi@parentprojectmd.org)

## Competing Interests

Dr. Cristian Ionita and Ms. Kathi Kinnett have no conflict of interest. Dr. Katherine Mathews has read the journals policy and has the following conflicts: she has been a site investigator for Duchenne clinical trials conducted by the following companies: Sarepta Therapeutics, Pfizer, Santhera, PTC, BMS and Italfarmaco.

## Data Availability Statement

All relevant data are within the paper.

## References

[1] Romitti PPS, Mathews K, et al.; Centers for Disease Control and Prevention (CDC). Prevalence of Duchenne/Becker muscular dystrophy among males aged 5-24 years: four states, 2007. MMWR Morb Mortal Wkly Rep 2009;58:1119–1122. 19834452

[2] Rangel, Vanessa, Ann S. Martin, and Holly L. Peay. "DuchenneConnect Registry Report." PLoS currents 4 (2012). 10.1371/currents.RRN1309 PMC3299489 22453902

[3] Ciafaloni, Emma, et al. "Delayed diagnosis in duchenne muscular dystrophy: data from the Muscular Dystrophy Surveillance, Tracking, and Research Network (MD STARnet)." The Journal of pediatrics 155.3 (2009): 380-385. 10.1016/j.jpeds.2009.02.007 PMC5884059 19394035

[4] Schram G, Fournier A, Leduc H, Dahdah N, Therien J, Vanasse M, Khairy P. All-cause mortality and cardiovascular outcomes with prophylactic steroid therapy in Duchennemuscular dystrophy. J Am Coll Cardiol 2013;61:948–954. 10.1016/j.jacc.2012.12.008 23352781

[5] Schram G, Fournier A, Leduc H, Dahdah N, Therien J, Vanasse M, Khairy P. All-cause mortality and cardiovascular outcomes with prophylactic steroid therapy in Duchennemuscular dystrophy. J Am Coll Cardiol 2013;61:948–954. 10.1016/j.jacc.2012.12.008 23352781

[6] Prior, T.W. and S.J. Bridgeman, Experience and strategy for the molecular testing of Duchenne muscular dystrophy. J Mol Diagn, 2005. 7(3): p. 317-26. 10.1016/S1525-1578(10)60560-0 PMC1867542 16049303

[7] Nichols, B., S. Takeda, and T. Yokota, Nonmechanical Roles of Dystrophin and Associated Proteins in Exercise, Neuromuscular Junctions, and Brains. Brain Sci, 2015. 5(3): p. 275-98. 10.3390/brainsci5030275 PMC4588140 26230713

[8] Allen, D.G., N.P. Whitehead, and S.C. Froehner, Absence of Dystrophin Disrupts Skeletal Muscle Signaling: Roles of Ca2+, Reactive Oxygen Species, and Nitric Oxide in the Development of Muscular Dystrophy. Physiol Rev, 2016. 96(1): p. 253-305. 10.1152/physrev.00007.2015 PMC4698395 26676145

[9] Hoffman, E.P., R.H. Brown, Jr., and L.M. Kunkel, Dystrophin: the protein product of the Duchenne muscular dystrophy locus. Cell, 1987. 51(6): p. 919-28. 10.1016/0092-8674(87)90579-4 3319190

[10] https://www.fda.gov/Drugs/DevelopmentApprovalProcess/DrugInnovation/ucm534863.htm

